# Potential preventive and therapeutic effect of Chinese herb rhubarb (da huang) for intensive care unit/pediatric intensive care unit gastrointestinal failure patients: A protocol for systematic review

**DOI:** 10.1097/MD.0000000000020188

**Published:** 2020-05-15

**Authors:** Wei Jin, Xiaohui Guo, Qingjie Li, Xiaoqiong Luo, Juan Zhong, Yang Song, Weiwei Hou, Yucui Guo, Yiwei Li, Junping Luo

**Affiliations:** a Hospital of Chengdu University of Traditional Chinese Medicine, Chengdu, Sichuan; b Chongqing Traditional Chinese Medicine Hospital, Chongqing; c The Second Hospital of Sichuan of TCM, Chengdu, Sichuan, China.

**Keywords:** da huang, gastrointestinal failure, intensive care unit/pediatric intensive care unit

## Abstract

**Introduction:**

The Chinese herb da huang (DH) (Rhubarb) is commonly used for GIF intensive care unit (ICU)/pediatric intensive care unit (PICU) gastrointestinal failure (GIF) patients in China. However, the potential preventive and therapeutic effect of DH in these patients has not yet been studied systematically.

**Objectives:**

The aim of this study was to evaluate the preventive and therapeutic effects of DH in treating ICU/PICU GIF patients with the most recent evidence.

**Methods:**

We systematically searched 7 databases from inception to March 30, 2018. RevMan 5.3 software was used to perform a meta-analysis. GRADE methodology was applied to evaluate the quality of evidence for each outcome. The review protocol was registered on PROSPERO (CRD42018092710) in advance.

**Results:**

Seven studies comprising 788 pediatric or adult participants were included in this analysis. Three indicators, including GIF occurrence rates (gastrointestinal mucosal hemorrhage, enteroplegia), multiple organ dysfunction syndrome (MODS)-related items (occurrence rates of MODS, mortality rates of MODS) and duration in the ICU was analyzed. The GIF occurrence rate meta-analysis result was (RR 0.47, CI 95% 0.37-0.60; *P* = .95); MODS related items indicator result was (RR 0.44, CI 95% 0.33–0.59; *P* = .41); ICU duration ICU result was (RR -2.87, CI 95% -3.53--2.21; *P* = .40). The safety of Chinese herb DH (Rhubarb) remains unclear.

**Conclusion:**

Current evidence suggests that the Chinese herb rhubarb (DH) powder combined with Western medicine was inferior to Western medicine alone in terms of preventive and therapeutic effects in ICU/PICU patients in terms of decreasing GIF occurrence rates (gastrointestinal mucosal hemorrhage and enteroplegia), occurrence rates of MODS, mortality from MODS, and shortened duration time in the ICU/PICU. However, larger sample sizes and rigorously-designed studies are necessary to conclusively determine the association between DH powder and outcomes in ICU/PICU GIF patients.

## Abstract 2

Supplemental Digital Content is available in the text

## 1 Introduction

Serious complications are often critical factors with respect to mortality of intensive care unit patients. Gastrointestinal failure (GIF), vital to the prognosis of critically ill patients in the intensive care unit, commonly complicates infections, severe trauma, shock and other pathological conditions. The systemic inflammatory response syndrome and multiple organ dysfunction syndrome (MODS) are common consequences.^[1,2,3,4,5]^ Therefore, it is a particularly important to prevent and treat GIF in intensive care unit (ICU) patients.^[6]^

A number of clinical studies have appeared recently in China to evaluate the Chinese herb rhubarb (da huang) as an adjuvant therapy for prevention and treatment GIF in ICU patients.^[7,8,9,10]^ Da huang (DH) has been reported to function as a gastrointestinal adhesion barrier protector, a promoter of gastrointestinal peristalsis,^[11]^ an alleviator of inflammatory reaction,^[12]^ and a reducer of gastrointestinal mucosa permeability.^[13]^ Based on these studies, DH has been used for GIF in ICU patients. However, none of these studies was a review of DH as an experimental intervention for ICU/pediatric intensive care unit (PICU) GIF patients. The evidence basis for the preventive and therapeutic efficacy of DH in this context has not been established to date. Therefore, the aim of this review was to critically and systematically assess the current state of evidence from RCTs on the use of DH in ICU/PICU GIF patients according to the Cochrane Handbook of meta-analysis.

## 2 Methods

### 2.1 Protocol registration

The review protocol was registered in advance on PROSPERO on date of April,4,2018, ID# CRD42018092710.

### 2.2 Search strategy

A systematic search for DH for ICU/PICU GIF trials was performed on March 30, 2018. All published and ongoing RCTs were searched. The languages were limited to Chinese or English.

### 2.3 Electronic searches

A total of seven databases were searched, including PubMed (1992 to March 30, 2018), EMBASE (Excerpta Medical Database) (1992 to March 30,2018), Cochrane Library (Issue 9 of March 30, 2018), Chinese Cochrane Center's Controlled Trials Register platform (up to March 30, 2018), Wanfang Chinese Digital Periodical and Conference Database (1997 to March 30, 2018), China National Knowledge Infrastructure Database (1992 to March 30, 2018), and the VIP Chinese Science and Technique Journals Database (1992 to March 30, 2018). In addition, the Chinese Clinical Trial Registry Center was searched for ongoing trials. In order to ensure complete searches for all trials, we checked references of relevant identified publications.

The search terms were as follows:

#1 Da-huang

#2 Da huang

#3 Rhubarb

#4 DH

#5 Chinese Herbal Medicine

#6 integrated Chinese and Western medicines

#7 integrated traditional and Western medicine

#8 #1-#7/OR

#9 PICU

#10 Intensive Care Unit

#11 ICU

#12 gastrointestinal failure

#13 GIF

#14 #9-#13/OR

#15 #8AND#14

### 2.4 Study selection criteria

This systematic review was designed to evaluate the preventive and therapeutic effect of DH for ICU/PICU patients of GIF. Studies were considered if they recruited ICU/PICU GIF patients without classification. Included studies were RCTs comparing nasal administration of DH powder to placebo or Western medical therapy or comparing the combination of DH and Western medicine (WM) to WM alone. If DH was used together with other Chinese herbs in a study, the study was excluded due to the difficulty of estimating the efficacy of DH in GIF. Observational studies, case reports, case series, qualitative studies, uncontrolled studies, and studies with no randomization-control design were excluded.

Studies were included if they reported one of the following predefined outcomes: occurrence rate of GIF, often reported as gastrointestinal mucosal hemorrhage and enteroplegia occurrence rates; occurrence rates or mortality rates of MODS; secondary outcomes were duration time of stay in ICU/PICU and adverse events (AEs).

### 2.5 Study selection, data extraction, and quality assessment

Study selection. After 2 authors (WJ, QL) scanned all titles and abstracts, a judgment was made regarding whether the trials met our inclusion criteria. Full-text screening was the next step, accomplished by 2 authors (JZ, YS). If there were conflicts, they were resolved by consensus.

Data extraction and management. Raw data of included papers included study identification, details regarding first authors and publication years, design details of the original study (duration, interventions for both groups, outcome measures, balance report of baseline, and randomization method and others). These were separately extracted by 2 authors (XL,YL).

### 2.6 Assessment of risk of bias

The methodological quality of the included RCTs was assessed according to the Cochrane Handbook for Systematic of Interventions. The latest version of this instrument was updated in March 2011, version 5.1.0 (http://www.handbook.cochrane.org).^[14]^ Risk of bias items included the following: randomization sequence generation, allocation concealment, blinding of participants or healthcare providers, detection bias, incompleteness bias, reporting bias, and other bias. Three options were considered: Low risk, high risk, and unclear. We defined other bias as trials possibly sponsored by drug companies and trials whose baseline characteristics were not similar among various intervention groups. Publication bias examination by funnel plots was not performed because there were fewer than 10 trials reporting primary outcomes.

### 2.7 Statistical analysis

Review Manager (RevMan) software version 5.3 was used to pool data and to execute the meta-analysis. GRADE software was the only quality evaluation tool used to demonstrate GRADE evidence ratings. According to the Cochrane Handbook for Systematic of Interventions, risk ratio (RR) was chosen for evaluation of dichotomous data. Mean difference (MD) was chosen for variable data. Confidence interval (CI) was set at 95%, and *P* < .05 was defined as statistically significant. *I*^2^ values were used to assess inter-study heterogeneity. According to the Cochrane Handbook, when *I*^2^ > 75%, considerable heterogeneity was confirmed, whereupon a random effects model would be applied. We pooled trials when the intervention form of those studies was adequately similar. Specific subgroups were analyzed according to similar intervention forms or similar designs.

## 3 Results

### 3.1 Study description

#### 3.1.1 Search results

One hundred and eleven trials were initially identified according to our protocol search strategy. No unpublished or ongoing studies were found. After titles, abstracts, and keywords were reviewed, 19 duplicated texts were excluded and 78 papers were excluded for failure to conform to inclusion criteria. Therefore 14 studies initially appeared to meet our inclusion criteria. After the full texts were read, seven studies were excluded because they were not true RCTs,^[15,16,17,18]^ they were duplicate publications^[19]^ or herbal medicine formulas were combined in the treatment group interventions.^[20,21]^ Seven studies finally met our inclusion criteria.^[13,22,23,24,25,26,27]^

The study selection process is outlined in Figure S1.

### 3.2 Patients

The 7 RCTs included 788 pediatric and adult ICU patients presenting with acute illnesses (pancreatitis, infectious shock, severe pneumonia, and others). There were 449 PICU and 339 ICU patients. There 443 males and 345 females.

### 3.3 Interventions

We compared raw rhubarb powder with conventional medicine or placebo regimens. There were 411 patients in the raw rhubarb powder (nasal administration) combined with WM group, and 377 patients in the WM alone group. The WM used in the 2 groups of 1 study was consistent. Five RCTs reported 3 to 7 day treatment courses (23–25,27), and 2 RCTs failed to mention duration of therapy.

### 3.4 Outcome measures

Trials were required to include as outcome measures either relief of symptoms or assessment of the efficacy of raw rhubarb powder for improvement of symptoms. Therefore, GIF occurrence rate (gastrointestinal mucosal hemorrhage, enteroplegia), MODS-related items (occurrence rate of MODS, mortality rate of MODS) would be the primary outcomes. Secondary outcomes included ICU duration and adverse events.

### 3.5 Included studies

We pooled seven Chinese-language trials including 788 PICU/ICU patients aged 1 day-83 years old.^[13,22,23,24,25,26,27]^ These trials were published between 2005 and 2011. All studies evaluated raw rhubarb powder + WM vs WM. Based on consistency of measurement of effective outcome rates in all 7 studies, GIF occurrence rate (gastrointestinal mucosal hemorrhage, enteroplegia) and MODS-related items (occurrence rate of MODS, mortality rate of MODS) were regarded as the most important outcome measures in all trials. Then, ICU duration was analyzed. Adverse events were not analyzed as they were not mentioned in any of the 7 trials. Characteristics of the seven included studies are displayed in Table S1.

### 3.6 Risk of bias

#### 3.6.1 Allocation (selection bias)

Seven studies were designed as randomized controlled trials, however, no study mentioned details of participants randomization method or details of patient allocation techniques. Hence, a high risk of bias could be ascribed to these trials.

#### 3.6.2 Blinding (performance bias and detection bias)

No blinding method was mentioned in any of the seven included studies. This might present a high risk of performance and detection bias.

#### 3.6.3 Incomplete outcome data (attrition bias)

None of the included studies reported information regarding sample size calculation. No study directly provided information regarding cases lost to follow-up or study withdrawals. Therefore, the risk of incomplete outcome data bias of those trials was unclear.

#### 3.6.4 Selective reporting (reporting bias)

None of the 7 included trials noted the protocol, and none of the trials declared a clinical trial registration number. Therefore, selective reporting bias is unclear.

#### 3.6.5 Other potential sources of bias

Although no signs of pharmaceutical company support were found in these studies. Three studies (Lu,^[22]^ Wang,^[24]^ and Zhang^[26]^) noted no statistical differences of balanced reporting at baseline. Therefore, other bias of this paper was low and others were unclear.

The risk of bias graph is displayed in Fig. S2 and the risk of bias summary is shown in Fig. S3.

#### 3.6.6 Effects of interventions

Seven studies comprising 788 pediatric or adult participants were included in this review. We analyzed 3 indicators: GIF occurrence rate (gastrointestinal mucosal hemorrhage, enteroplegia), MODS-related items (occurrence rate of MODS, mortality rate of MODS) and ICU duration.

The GIF occurrence rate of the seven RCTs is shown in Fig. S4. MODS-related items are shown in Figure S5. ICU duration is shown in Figure S6.

#### 3.6.7 GIF occurrence rate

Five studies^[13,22,23,25,27]^ reported gastrointestinal mucosal hemorrhage occurrence rates in both groups, and four studies^[13,23,25,27]^ reported enteroplegia occurrence rates in both groups. We pooled all data and the result revealed (RR 0.47, CI 95% 0.37–0.60; *P* = .95; Figure S4), with benefit for the experimental group.

#### 3.6.8 MODS-related items

Three studies reported MODS occurrence rates for both groups, and 3 studies^[23,25,26]^ reported MODS mortality rates for both group. We pooled all data and the results revealed (RR 0.44, CI 95% 0.33–0.59; *P* = .41; Fig. S5) with benefit for the experimental group.

#### 3.6.9 ICU duration

Three studies^[13,25,26]^ reported ICU duration for both groups. All data were pooled and analyzed, and the result was (RR -2.87, CI 95% -3.53--2.21; *P* = .40; Figure S6). This is suggested that DH combined with WM was superior to WM alone for prevention and treatment of GIF patients in the pediatric and adult ICU.

#### 3.6.10 Adverse events

Adverse events were not analyzed because none of the studies reported this information. Hence, we cannot assess adverse events related to DH vs WM in GIF patients in the ICU.

#### 3.6.11 Quality of evidence

The quality of evidence for outcome measures, according to the GRADE system are displayed in Table S1. The quality of evidence evaluation returned 6 low results and 1 very low result.

## 4 Discussion

### 4.1 Overview of findings

To the best of our knowledge, this is the first systematic review of potential preventive and therapeutic effects of DH in GIF patients in the ICU. Seven trials were included in our review. We found that DH powder combined with WH was inferior to WM alone in terms of prevention and therapy for GIF in ICU/PICU patients, in terms of occurrence rates of GIF (gastrointestinal mucosal hemorrhage, enteroplegia), occurrences rate of MODS, mortality rates of MODS, and shortened duration time in the ICU/PICU (Fig. 4, 5, 6). There were varying simple sizes and poor quality of experimental designs according to the GRADE methodology (Table S2). In these 7 studies, none applied validated questionnaires or scales to assess the improvement of the quality of life. In addition, there were no evaluations of the safety of DH powder.

### 4.2 Quality of evidence

All included studies were prospective, randomized, placebo-controlled studies. However, no studies mentioned the method of randomization. No study stated whether the design was double-blinded. Therefore, there was a potential risk of measurement and implementation bias. No trial mentioned allocation concealment or any concealment method. It was not clear whether incomplete outcomes data were adequately addressed, as no trial reported drop-out rates. Therefore, the risk of incomplete outcome data bias was unclear. Three studies (22,24,26) made reference to balance report of baseline, therefore other bias in these papers was low and in the others, it was unclear. Therefore, according to the GRADE system, the quality of evidence for outcome measurements was low for six papers and very low for 1 paper.

In terms of clinical trial reporting, none of the included studies reported all items recommended by CONSORT 2010 and its Extension for Herbal Intervention.^[28,29]^ In addition, in the absence of trial registration information or published protocols, evaluation of selective reporting bias was not applicable to any of included studies.

### 4.3 Potential biases in the review process

Conclusions from this review were drawn from seven included trials due to the absence of ongoing trials; there were a limited number of patients. Studies with larger numbers of patients and high-quality designs should be performed in the future. Another key issue was that the treatment course for assessing outcome varied from trial to trial. The treatment course in these studies varied from 3 days to 1 week, or was not mentioned. Several Western medications for treatment of the original disease in groups were designated “Western medicine therapy”. However, these interventions differed because the primary disease differed. All included studies not mentioned whether there was any adverse effect by the WM on GIF or ICU duration when assessed the combination (powder + WM) effect because the sequence/order of combination drugs will also signify whether the powder was effective or not compared to WM. These critical differences might be a factor leading to bias.

## 5 Conclusion

We found that DH powder combined with WM was inferior to WM alone in terms of prevention and treatment in ICU/PICU patients in terms of GIF occurrence rates (including gastrointestinal mucosal hemorrhage and enteroplegia), occurrence rates of MODS, mortality rates of MODS, and ICU/PICU duration. Safety DH powder is unknown. All included studies had poor quality and design limitations.

Therefore, larger sample sizes and rigorously-designed studies are necessary to determine conclusively a definitive association between Chinese herb Rhubarb (DH) powder in ICU/PICU GIF patients.

### 5.1 Implications for practice

There is an uncertain recommendation for the use of Chinese herb Rhubarb (DH) powder for GIF patients in the ICU/PICU because of unclear safety.

### 5.2 Implications for research

There is an urgent need for double-blind, prospective, randomized, placebo-control trials of DH powder as adjuvant treatment for ICU/PICU GIF patients. Long-term follow-up efficacy and safety is required in those future studies. More indicators related to its efficacy in GIF, including borborygmi, abdominal distention, and objective items such as intra-abdominal pressure, gastric acid, and others are possible recommend outcomes.

## Author contributions

Conceived and designed the experiments: WJ, XG, QL and JZ. Performed the experiments: YS, WJ. Analyzed the data: JZ, XL. Contributed reagents/materials/analysis tools: YL, WH. Wrote the paper: WJ, JZ.

**Conceptualization:** Wei Jin.

**Data curation:** Qingjie Li.

**Investigation:** Yucui Guo, Yiwei Li, Junping Luo.

**Methodology:** Wei Jin.

**Software:** Xiaoqiong Luo, Juan Zhong, Yang Song.

## Supplementary Material

Supplemental Digital Content

## Supplementary Material

Supplemental Digital Content

## Supplementary Material

Supplemental Digital Content

## Supplementary Material

Supplemental Digital Content

## Supplementary Material

Supplemental Digital Content

## Supplementary Material

Supplemental Digital Content

## Supplementary Material

Supplemental Digital Content

## Supplementary Material

Supplemental Digital Content
