# Supplementary material for: Potential preventive and therapeutic effect of Chinese herb rhubarb (da huang) for intensive care unit/pediatric intensive care unit gastrointestinal failure patients: A protocol for systematic review
Source: Medicine (Baltimore). 2020 May 15;99(20):e20188. doi: 10.1097/MD.0000000000020188 (PMC7254143; doi:10.1097/MD.0000000000020188)
Supplement: Supplemental Digital Content [file medi-99-e20188-s001.docx]

Table S1 The quality of evidence for outcome measures of 7 studies.

| **Quality assessment** | | | | | | | | | **Summary of findings** | | | | | **Importance** |
| --- | --- | --- | --- | --- | --- | --- | --- | --- | --- | --- | --- | --- | --- | --- |
|  |  |  |  |  |  |  |  |  | **No of patients** | | **Effect** | | **Quality** |  |
| **No of studies** | **Design** | **Limitations** | **Inconsistency** | | **Indirectness** | | **Imprecision** | **Other considerations** | **Outcome measures** | **control** | **Relative (95% CI)** | **Absolute** |  |  |
| **GIF** | | | | | | | | | | | | | | |
| 5 | randomized trials^1^ | serious^2^ | no serious inconsistency^3^ | | | serious^4^ | no serious imprecision^5^ | none^6^ | 84/634 (13.2%) | 162/572 (28.3%) | RR 0.47 (0.37 to 0.6) | 150 fewer per 1000 (from 113 fewer to 178 fewer) |  LOW | IMPORTANT^7^ |
|  |  |  |  |  |  |  |  |  |  | 30% |  | 159 fewer per 1000 (from 120 fewer to 189 fewer) |  |  |
| **GIF - occurrence rate of Gastrointestinal mucosal hemorrhage** | | | | | | | | | | | | | | |
| 5 | randomized trials^1^ | serious^2^ | | no serious inconsistency^3^ | | serious^4^ | no serious imprecision^5^ | none^6^ | 53/348 (15.2%) | 104/317 (32.8%) | RR 0.47 (0.35 to 0.63) | 174 fewer per 1000 (from 121 fewer to 213 fewer) |  LOW | IMPORTANT^7^ |
|  |  |  |  |  |  |  |  |  |  | 31.2% |  | 165 fewer per 1000 (from 115 fewer to 203 fewer) |  |  |
| **GIF - occurrence rate of enteroplegia** | | | | | | | | | | | | | | |
| 4 | randomized trials^1^ | serious^2^ | | no serious inconsistency^3^ | | serious^4^ | no serious imprecision^5^ | none^6^ | 31/286 (10.8%) | 58/255 (22.7%) | RR 0.48 (0.32 to 0.71) | 118 fewer per 1000 (from 66 fewer to 155 fewer) |  LOW | IMPORTANT^77^ |
|  |  |  |  |  |  |  |  |  |  | 22% |  | 114 fewer per 1000 (from 64 fewer to 150 fewer) |  |  |
| **MODS** | | | | | | | | | | | | | | |
| 5 | randomized trials^1^ | serious^2^ | | no serious inconsistency^3^ | | serious^4^ | no serious imprecision^5^ | none^6^ | 98/481 (20.4%) | 199/429 (46.4%) | RR 0.44 (0.33 to 0.59) | 260 fewer per 1000 (from 190 fewer to 311 fewer) |  LOW | IMPORTANT^7^ |
|  |  |  |  |  |  |  |  |  |  | 51.3% |  | 287 fewer per 1000 (from 210 fewer to 344 fewer) |  |  |
| **MODS - occurrence rate of MODS** | | | | | | | | | | | | | | |
| 3 | randomized trials^1^ | serious^2^ | | no serious inconsistency^3^ | | serious^4^ | no serious imprecision^5^ | none^6^ | 72/246 (29.3%) | 125/215 (58.1%) | RR 0.51 (0.41 to 0.63) | 285 fewer per 1000 (from 215 fewer to 343 fewer) |  LOW | IMPORTANT^7^ |
|  |  |  |  |  |  |  |  |  |  | 59.7% |  | 293 fewer per 1000 (from 221 fewer to 352 fewer) |  |  |
| **MODS - Mortality rate of MODS** | | | | | | | | | | | | | | |
| 3 | randomized trials^1^ | serious^2^ | | no serious inconsistency^3^ | | serious^4^ | no serious imprecision^5^ | none^6^ | 26/235 (11.1%) | 74/214 (34.6%) | RR 0.37 (0.18 to 0.76) | 218 fewer per 1000 (from 83 fewer to 284 fewer) |  LOW | IMPORTANT^7^ |
|  |  |  |  |  |  |  |  |  |  | 34.4% |  | 217 fewer per 1000 (from 83 fewer to 282 fewer) |  |  |
| **Duration time of stay in ICU (Better indicated by lower values)** | | | | | | | | | | | | | | |
| 3 | randomized trials^1^ | serious^2^ | | serious^3^ | | serious^4^ | no serious imprecision^5^ | none^6^ | 116 | 105 | - | MD 2.87 lower (3.53 to 2.21 lower) | VERY LOW | IMPORTANT^7^ |

^1^ Some studies had a high risk of bias due to their methodology
^2^ The study had performance bias and detection bias
^3^ Total number of events was less than 300
^4^ All studies were from China
^5^ Only one study or two studies
^6^ Further research is needed
^7^ Uncertain about the estimate
