# Supplementary figures and images for: Potential preventive and therapeutic effect of Chinese herb rhubarb (da huang) for intensive care unit/pediatric intensive care unit gastrointestinal failure patients: A protocol for systematic review
Source: Medicine (Baltimore). 2020 May 15;99(20):e20188. doi: 10.1097/MD.0000000000020188 (PMC7254143; doi:10.1097/MD.0000000000020188)

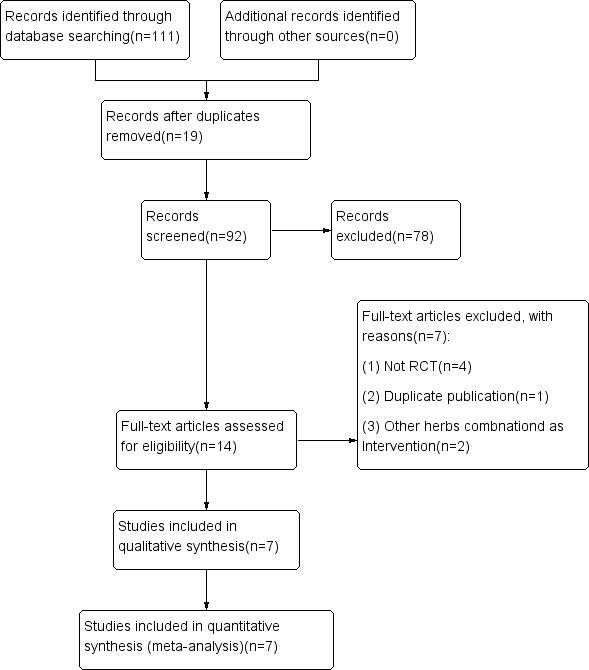

Supplement: Supplemental Digital Content [file medi-99-e20188-s003.jpg]

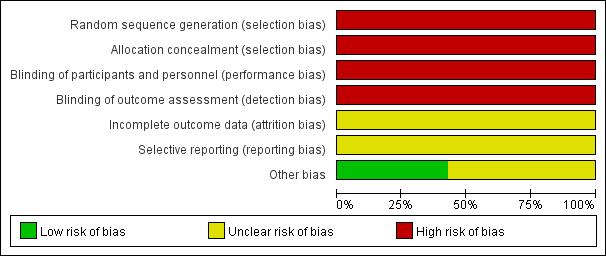

Supplement: Supplemental Digital Content [file medi-99-e20188-s004.jpg]

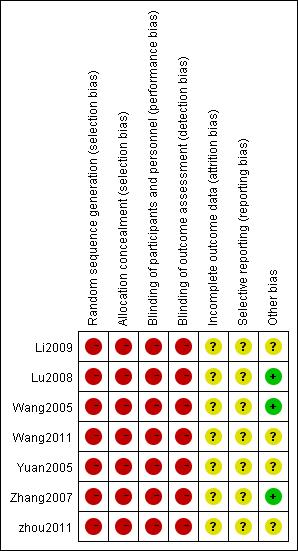

Supplement: Supplemental Digital Content [file medi-99-e20188-s005.jpg]

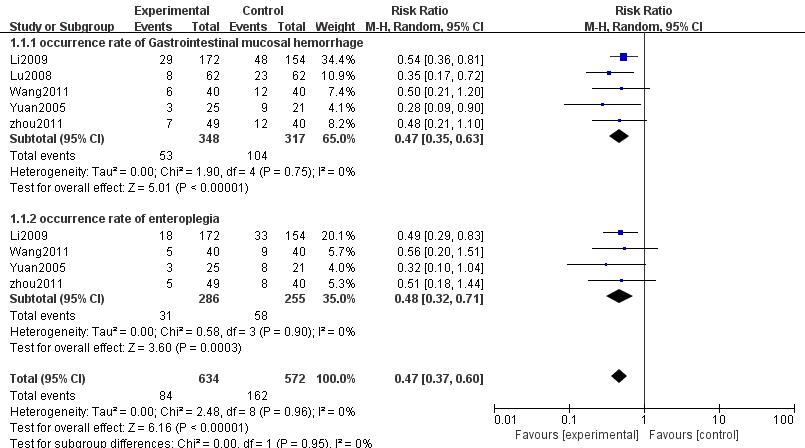

Supplement: Supplemental Digital Content [file medi-99-e20188-s006.jpg]

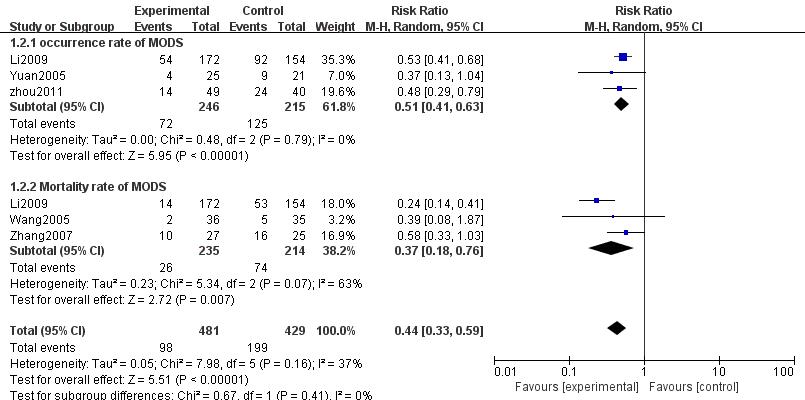

Supplement: Supplemental Digital Content [file medi-99-e20188-s007.jpg]

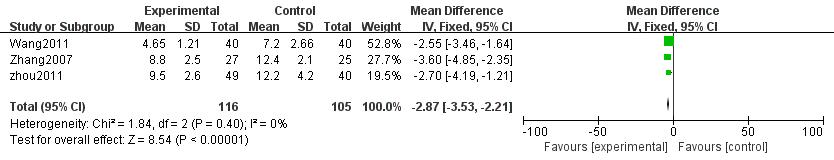

Supplement: Supplemental Digital Content [file medi-99-e20188-s008.jpg]
